# Response of Typical Shrubs Growth and Soil Nutrients to Graphene Addition in Impoverished Land of the Ulan Buh Desert

**DOI:** 10.3390/plants13223214

**Published:** 2024-11-15

**Authors:** Ren Mu, Jun Qiao, Chuijiu Kong, Xuting Hao, Guangfu Xu, Jingfu Han, Xinle Li

**Affiliations:** 1 Experimental Center of Desert Forestry, Chinese Academy of Forestry, Dengkou 015200, China; mr060814@126.com (R.M.); 15849890049@163.com (C.K.); haoxuting123@126.com (X.H.); 15164895533@163.com (G.X.); 13947819189@163.com (J.H.); 2 Engineering Research Center of Coal-Based Ecological Carbon Sequestration Technology of the Ministry of Education, Shanxi Datong University, Datong 037009, China; qiaojun_nk@163.com

**Keywords:** desert areas, graphene, shrubs, plant morphology, soil nutrients

## Abstract

Graphene can promote plant growth and improve soil conditions, but its effectiveness in enhancing infertile soils in arid regions remains unclear. This study selected three typical shrubs from the Ulan Buh Desert *Nitraria tangutorum*, *Xanthoceras sorbifolium*, and *Amygdalus mongolica* as research subjects. Five graphene addition levels were set: 0 mg/L (C0), 25 mg/L (C1), 50 mg/L (C2), 100 mg/L (C3), and 200 mg/L (C4).A pot experiment was conducted in June 2023 to investigate the effects of graphene addition on shrub growth and soil nutrients. The results showed that the optimal graphene addition levels for *A. mongolica*, *X. sorbifolium*, and *N. tangutorum* were C2, C2, and C3, respectively. Compared with the control, the total biomass of the different shrubs increased by 185.31%, 50.86%, and 161.10%, respectively. However, when the graphene addition exceeded the optimal level, shrub biomass showed a decreasing trend with increasing graphene concentration. Total shrub biomass was positively correlated with soil available nitrogen and potassium, while redundancy analysis indicated that soil organic matter was the primary factor influencing shrub growth. This suggests that graphene promotes shrub growth by affecting soil organic matter and available nutrients. Therefore, graphene addition can enhance soil fertility in barren lands in arid regions and significantly promote shrub growth. However, due to soil leaching effects, this growth-promoting effect may decrease over time.

## 1. Introduction

Desertification is a major global ecological problem that threatens human survival and development. China, one of the countries most severely affected by desertification, has vast expanses of desertified land, concentrated regional distribution. Approximately 84% of desertified land is located in the northwest [1]. For decades, China has attach importance to desertification prevention and control. Through a series of robust measures has successfully curbed the expansion of desertified areas and significantly improved the ecological environment [2]. However, desert regions are often located in areas with frequent wind and sand activity, there are many strong winds and sparse vegetation. Soil nutrients are relatively poor compared to other regions in China. The key issue of ecological governance and vegetation restoration in desert areas is how to improve soil fertility.

In recent years, soil amendments such as straw, biochar, and graphene have become key measures for improving soil quality [3,4]. ZHANG et al. [5] demonstrated that chicken manure biochar can enhance soil potential and promote plant root growth. Similarly, WANG et al. [6] found that straw incorporation into fields can increase microbial biomass, soil carbon, and nitrogen content, thereby enhancing maize photosynthetic efficiency and promoting crop growth. However, these soil amendments also present limitations. For instance, biochar, particularly due to its alkaline properties, is more effective in acidic soils but may reduce crop yields in alkaline soils [7,8]. The acquisition time and quantity of straw materials are often unpredictable, resulting in higher raw material costs, and straw’s low durability can result in rapid decomposition, compromising the persistence of soil organic matter [9]. Graphene, a nanocarbon material composed of carbon atoms arranged in a two-dimensional crystal lattice, contains functional groups that contribute to its high water dispersibility and conductivity. Its layered surface structure for effective adsorption of nutrient ions and water, providing soil with moisture retention and nutrient collection capabilities [10]. Compared to other soil amendments like straw and biochar, graphene offers a larger specific surface area and a higher concentration of functional groups, significant water and fertilizer retention effect. Consequently, graphene is being increasingly adopted in agricultural and forestry applications.

The addition of graphene can enhance water absorption by plant roots, increase the efficiency of soil nutrient utilization, stimulate plant cell metabolism, promote growth, and further improve plant stress resistance based on original trait basis [11,12,13]. LIU et al. [14] reported that graphene significantly affects the growth of *Lolium perenne* seedlings, with low concentrations promoting growth and high concentrations inhibiting it. ZHAO et al. [15] demonstrated that graphene enhances antioxidant enzyme activity in *Danfeng*, promotes lignin biosynthesis, and improves drought resistance. In addition to influencing plant physiology and phenotype, graphene can act as a fertilizer or soil nutrient enhancer. By adsorbing different ions in the soil, it aids in nutrient retention and improves soil physicochemical properties [16,17]. Wang et al. [18] found that graphene addition alters the average diameter and proportion of large soil aggregates, thereby increasing the availability of soil nutrients. In general, the addition of graphene at low concentrations promoted plant growth and improved soil environment, while high concentrations had adverse effects on plants and soil.

Due to, as a relatively new nanocarbon material, there is still debate over whether graphene can be directly added to soil as a “fertilizer.” Some scholars [19] argue that the oxygen-containing functional groups on graphene may undergo Fenton reactions with metal ions in the soil, converting some graphene into CO_2_ and potentially causing toxic effects. Nevertheless, not all C-C bonds in graphene break to form oxygen-containing groups, suggesting that the risk of such effects is limited. For example, Chung et al. [20] found that the addition of graphene would temporarily reduce soil enzyme activity, but it would recover after a short-term decline, and would not lead to the reduction of soil microbial biomass. Ren et al. [21] found that graphene had no significant effect on soil bacterial community, but only had a certain effect on some autotrophic nitrogen fixing bacteria. However, some scholars have found that graphene can be absorbed by plants and accumulated in vivo. For example, Chen et al. Labeled graphene in pea with ^13^C isotope. Graphene only existed in the root and significantly inhibited the photosynthesis of pea [22]. To sum up, graphene has certain toxicity to plants and soil. The degree of toxicity mainly depends on the nature, concentration and treatment time of graphene, and is also related to plant species and genotypes [23]. Therefore, the potential environmental hazards and ecological risks of graphene materials need to be fully studied and evaluated before they are applied to the agriculture and forestry industry. Strengthening the exploration of the safe use of graphene can provide scientific data and theoretical basis for its industrial application.

The soils in arid areas northwestern China, including chestnut calcareous soil, brown calcareous soil, and desert soil, are mostly alkaline and barren [24]. Ulan Buh Desert is located in the extremely dry and early inland area of northwest china. Its northeast is an oasis, the Yellow River is in the east, and the west and south belong to the desert transition zone. It has a special position in the arid area of Northwest China. At the same time, Ulan Buh Desert is an important experimental demonstration area for desertification control in china. Over the past decade, ecological restoration measures, such as reforestation and grassland restoration, have led to a positive trend of “overall improvement and accelerated improve” in the management of desertified land. However, the issue of soil fertility barrenness of desertified land has not fundamentally changed [25]. This study selected typical shrubs from the Ulan Buh Desert—*Nitraria tangutorum*, *Xanthoceras sorbifolium*, and *Amygdalus mongolica*. Exploration the growth trends of typical shrub seedlings in the Ulan Buh Desert and the changes in soil nutrient levels under different concentrations of graphene addition, based on the hypothesis that “graphene addition can promote the growth of typical shrub seedlings and enhance soil nutrient content.” The aim is to determine the optimal amount of graphene for difference shrub species, with the objective of improving soil nutrient deficiencies in the Ulan Buh Desert and providing theoretical support for ecological restoration efforts in challenging areas of this region.

## 2. Results

### 2.1. The Effect of Graphene Addition on the Morphological Characteristics of Different Shrubs

According to Table 1, compared to the control group, after 30 days of graphene addition, the plant height of *X. sorbifolium* and *N. tangutorum* increased significantly by 28.35% and 22.35%, respectively, under C1 treatment (*p* < 0.05). Under C4 treatment, the basal diameters of *A. mongolica* and N. tangutorum increased by 25.00% and 18.06%, respectively, compared to the control (*p* < 0.05). Additionally, the leaf width and length of *A. mongolica* and *X. sorbifolium* reached their maximum values under C2 treatment, significantly higher than the control (*p* < 0.05). After 60 days of graphene addition, the basal diameter and leaf width of *X. sorbifolium* were significantly greater under C3 and C2 treatments, increasing by 17.98% and 22.97%, respectively (*p* < 0.05). Under C2 treatment, the basal diameter of *N. tangutorum* increased by 39% compared to the control (*p* < 0.05). However, under C4 treatment, the leaf length of *X. sorbifolium* reached its minimum value, a 19.05% decrease compared to the control. After 90 days, treatment with C1 resulted in a significant 50.31% increase in the basal diameter of *A. mongolica* compared to the control (*p* < 0.05). Conversely, under C4 treatment, the leaf width of *N. tangutorum* and the leaf length of *X. sorbifolium* decreased by 18.96% and 12.18%, respectively, compared to their control groups (*p* < 0.05).

### 2.2. The Effect of Graphene Addition on Biomass of Different Shrubs

**Figure 1:**
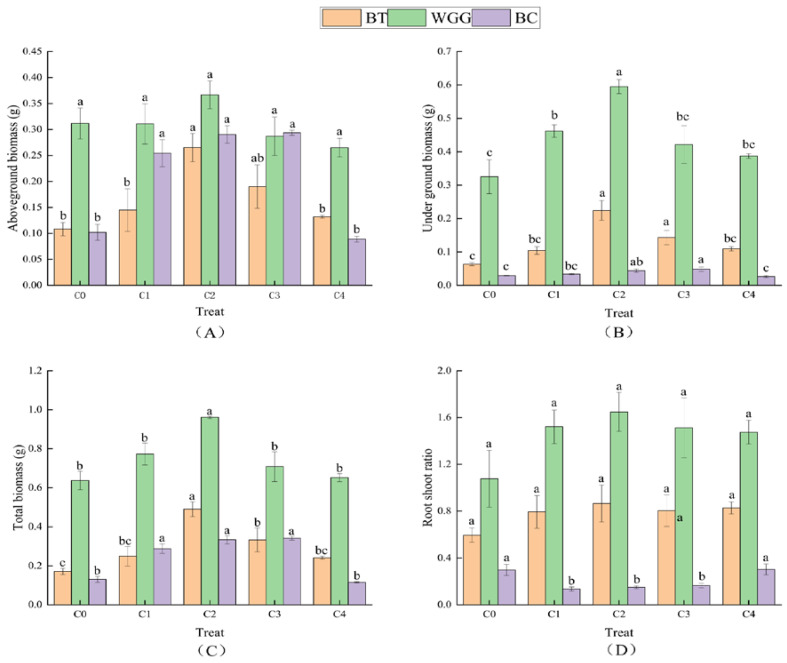
The effect of different levels of graphene addition on shrub biomass and root crown ratio. Note: Different small letters indicate significant differences between different treatments under the same plant (*p* < 0.05), the same below.

From Figure 1, it can be observed that, compared to the control, the C2 treatment resulted in a significant increase in the aboveground biomass, underground biomass, and total biomass of *A. mongolica* by 144.97%, 254.34%, and 185.31%, respectively (*p* < 0.05). Under the C3 treatment, the aboveground biomass, underground biomass, and total biomass of *N. tangutorum* increased significantly by 184.61%, 67.93%, and 161.10% compared to the control group (*p* < 0.05). For *X. sorbifolium*, there was no significant difference in aboveground biomass between the C2 treatment and the control, but the underground biomass and total biomass increased significantly by 82.91% and 50.86%, respectively (*p* < 0.05). Additionally, *A. mongolica* and *X. sorbifolium* reached their maximum root-to-shoot ratios under C2 treatment, whereas N. tangutorum exhibited significant reductions in root-to-shoot ratio under C1, C2, and C3 treatments by 54.41%, 49.39%, and 44.35%, respectively, compared to the control (*p* < 0.05).

### 2.3. The Effect of Graphene Addition on Nutrients in Different Shrubs Soil

As shown in Table 2, the total nitrogen and total potassium content in the soil surrounding *A. mongolica* increased significantly by 80.09% and 11.28%, respectively, under C3 and C2 treatments compared to the control (*p* < 0.05). Additionally, the available nitrogen and available potassium content in the soil increased significantly by 36.75% and 36.73%, respectively, under C2 treatment compared to the control (*p* < 0.05). The trends in total and available nutrients in the soil of *X. sorbifolium* were similar. Under C2 treatment, the total nitrogen and available nitrogen content increased significantly by 13.42% and 30.36%, respectively, compared to the control (*p* < 0.05). Similarly, the total phosphorus and available phosphorus content increased significantly by 12.50% and 17.25%, respectively (*p* < 0.05). In contrast, the organic matter and total nutrients in the soil surrounding *N. tangutorum* showed no significant response to graphene addition. However, the available nitrogen and available potassium content increased significantly by 53.20% and 47.40% under C3 and C2 treatments, respectively, compared to the control (*p* < 0.05).

### 2.4. Analysis of the Influencing Factors of Graphene Addition on the Growth of Different Shrubs and Soil Nutrients

Principal component analysis of 15 indicators related to shrub growth and soil nutrients under different treatments (Table 3) reveals that total shrub biomass, available soil phosphorus, shrub height, and basal diameter are the key indicators for analyzing the effects of graphene addition on shrub growth and soil nutrients, with a cumulative contribution rate of 68.99%.

**Figure 2:**
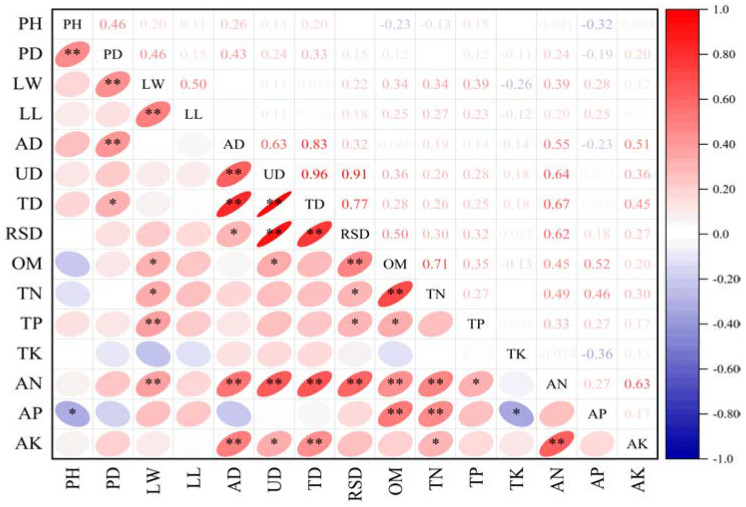
Correlation analysis between shrub growth indicators and soil nutrients at different levels of graphene addition. Note: PH: Plant height; PD: base diameter; LW: Leaf width; LL: Leaf length; AD: aboveground biomass; UD: Underground biomass; TD: Total biomass; RSD: Root to shoot ratio; OM: Organic matter; TN: Total nitrogen; TP: Total phosphorus; TK: Total potassium; AN: Available nitrogen; AP: Available phosphorus, AK: Available potassium, ** indicates a very significant level of *p* < 0.01, and * indicates a significant level of *p* < 0.05.

**Figure 3:**
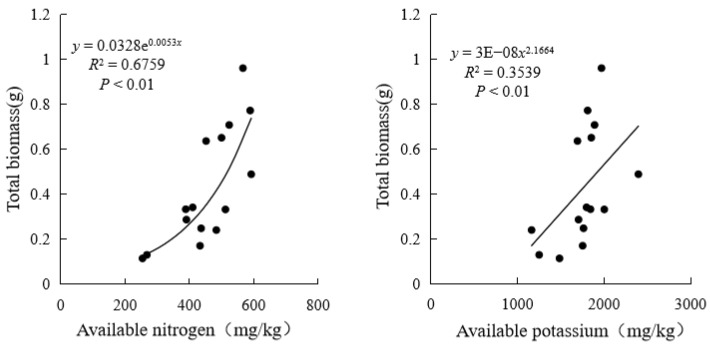
Linear fitting of total biomass of shrubs with different levels of graphene addition to soil available nitrogen and available potassium.

As shown in Figure 2, graphene addition resulted in a highly significant positive correlation between shrub height and basal diameter (*p* < 0.01), and a significant negative correlation with available soil phosphorus (*p* < 0.05). Basal diameter was highly significantly positively correlated with leaf width and aboveground biomass (*p* < 0.01) and significantly positively correlated with total biomass (*p* < 0.05). Total biomass exhibited a highly significant positive correlation with the root-to-shoot ratio, available nitrogen, and available potassium in the soil (*p* < 0.01). Additionally, principal component analysis indicated that total biomass is the primary indicator for evaluating the impact of graphene on shrub growth and soil nutrients. A linear fitting analysis of indicators significantly correlated with total biomass revealed a functional relationship between shrub biomass and soil nutrients. As shown in Figure 3, total shrub biomass exhibited an exponential relationship with available soil nitrogen and a power-law relationship with available soil potassium, both increasing as nutrient levels rose. These findings suggest that graphene addition promotes shrub biomass growth by enhancing soil nutrient content.

### 2.5. Analysis of Shrub Growth and Soil Nutrient Redundancy Under Graphene Addition

**Figure 4:**
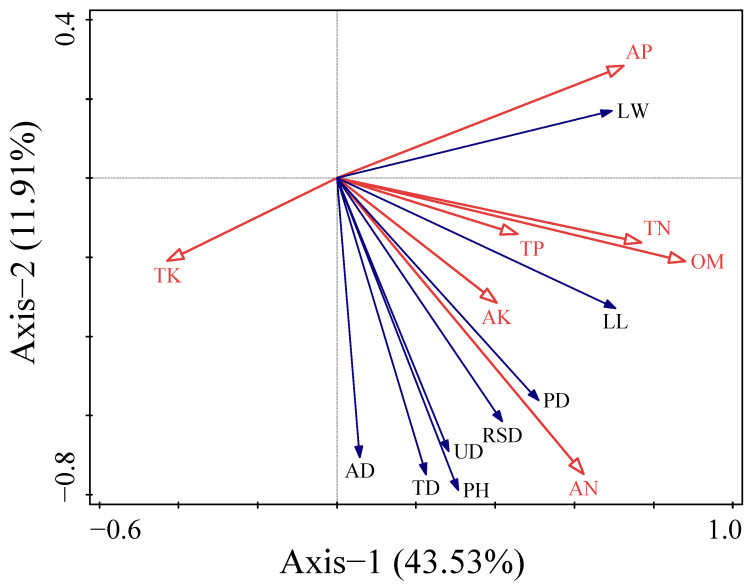
Correlation analysis of growth indicators and soil physicochemical properties of bromus inermis. Note: PH: Plant height; PD: base diameter; LW: Leaf width; LL: Leaf length; AD: aboveground biomass; UD: Underground biomass; TD: Total biomass; RSD: Root to shoot ratio; OM: Organic matter; TN: Total nitrogen; TP: Total phosphorus; TK: Total potassium; AN: Available nitrogen; AP: Available phosphorus, AK: Available potassium.

Redundancy analysis (RDA) results (Figure 4) show that soil nutrients accounted for 55.44% of the total variation in shrub growth indicators. The first and second axes of soil nutrients explained 43.53% and 11.91% of the variation in shrub growth indicators, respectively. The importance ranking of soil nutrients influencing shrub growth indicators was as follows: OM > AN > AP > TN > TK > AK > TP. Organic matter (OM) was identified as the key factor affecting shrub growth indicators, with an explanatory rate of 34.40% (F = 6.8, *p* < 0.01), and it exhibited a positive correlation with all shrub growth indicators (Table 4).

### 2.6. Cluster Analysis and Comprehensive Analysis of Different Shrub Growth and Soil Nutrients Under Graphene Addition

**Figure 5:**
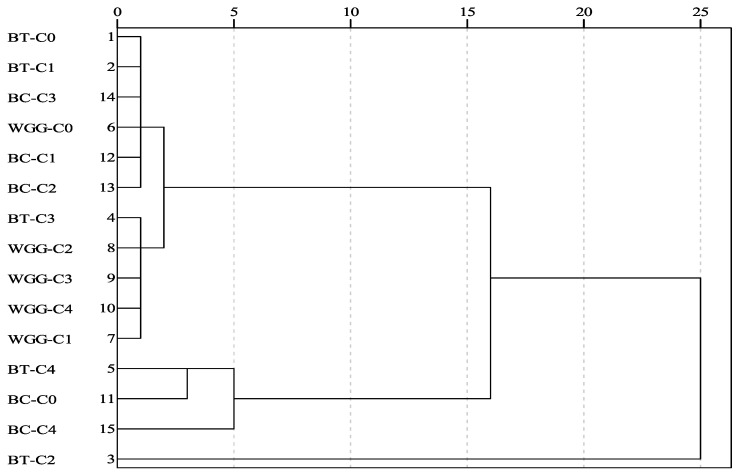
Cluster analysis of shrub growth indicators and soil nutrients at different levels of graphene addition.

Cluster analysis of the growth and soil nutrient indicators for three shrub species under five graphene treatment gradients (Figure 5) shows that, at an Euclidean distance of 10, the 15 treatments can be grouped into three clusters. The first cluster includes the BT-C2 treatment, characterized by large leaf width and length, higher biomass, and elevated soil nutrient content, indicating the best overall traits. The second cluster consists of the BT-C0, BT-C1, BC-C3, WGG-C0, BC-C1, BC-C2, BT-C3, WGG-C2, WGG-C3, WGG-C4, and WGG-C1 treatments, which are associated with average shrub growth and moderate soil nutrient content, reflecting relatively poorer overall traits. The third cluster includes the BT-C4, BC-C0, and BC-C4 treatments, where shrubs exhibited stunted growth, thinner basal diameters, and generally lower biomass and soil nutrient content, indicating the poorest overall traits. Based on the comprehensive grey relational analysis results, it was determined that the optimal treatment for *A. mongolica* and *X. sorbifolium* is C2, while for *N. tangutorum*, the optimal treatment is C3 (Table 5).

## 3. Discussion

### 3.1. The Effect of Different Levels of Graphene Addition on the Growth of Different Shrubs

As the application of graphene in the agricultural and forestry sectors gains increasing attention, its effects on plant growth have been widely studied, generally showing a trend of promoting growth at low concentrations and inhibiting growth at high concentrations. However, the response to graphene addition varies among plant species [12], graphene particle size [13], and plant growth patterns [26]. This study, which investigated the addition of graphene to different shrub species, demonstrates that graphene has a noticeable promotive effect on shrub growth traits. This may be attributed to graphene’s ability to stimulate the activation of quiescent cells by promoting auxin induction, accelerating cell division in stems and roots, and thereby enhancing plant growth [13]. Moreover, graphene’s large specific surface area, when adsorbed onto the surface of plant roots, can act as an ion transport channel, increasing nutrient absorption rates and further promoting growth [27]. Zhang et al. [28] found that graphene can improve photosynthesis by increasing stomatal density, stomatal conductance, and intercellular CO_2_ concentration, thereby enhancing nutrient content such as proteins and amino acids in leaves, which boosts the plant’s photosynthetic capacity and growth rate. However, other scholars [16,29] discovered that graphene does not necessarily increase photosynthesis but can significantly enhance plant antioxidant enzyme activity and indole-3-acetic acid (IAA) levels under drought stress. IAA is the most common natural auxin that regulates root structure and growth. Since roots are the main organs for nutrient absorption and utilization, they play a key role in the synthesis and accumulation of aboveground biomass, directly influencing plant growth and biomass production [30].

**Figure 6:**
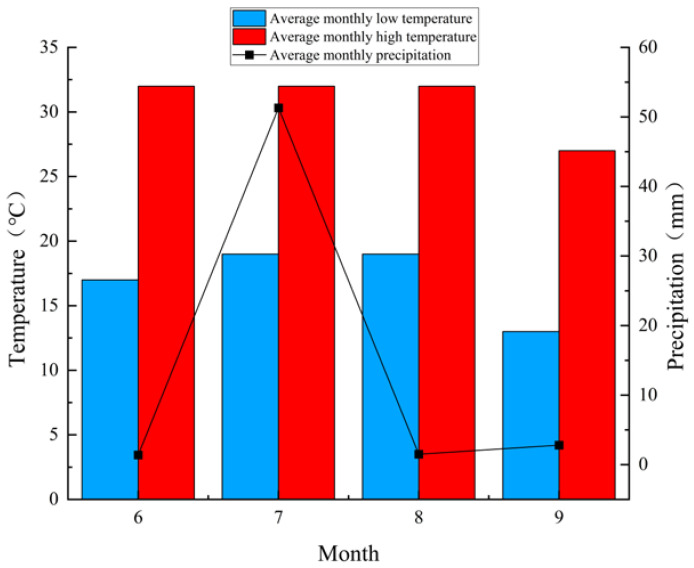
Temperature and precipitation changes from June to September in the study area.

The study found that after 90 days, the growth effects of the shrubs began to decline, which may be related to climatic factors. After entering September, temperatures began to drop (Figure 6), leading to a slowdown in the metabolic processes of the shrubs. Therefore, when investigating the effects of graphene addition on plant growth, it is also essential to consider the influence of environmental factors. Consistent with the conclusions of most researchers [31], this study found that moderate graphene addition significantly increases biomass in various shrubs. However, excessive graphene can have toxic effects on plant roots, altering their morphology, causing structural damage, reducing chlorophyll content, inhibiting growth, and inducing genetic toxicity or oxidative stress [32]. It is worth noting that the morphological characteristics of different shrubs respond differently to graphene. This study found that after 30 days of graphene addition, the leaf width and length of all three shrubs reached their maximum values under the C2 treatment, while height and basal diameter exhibited varied responses to graphene. Additionally, the shrub response to different concentrations of graphene changed over time. For example, the basal diameter of *A. mongolica* reached its maximum value under C4 treatment after 30 days, but after 90 days, the maximum was observed under C1 treatment. In summary, the effect of graphene on plant growth and development depends on the specific characteristics of the plant [33]. Therefore, precise concentration gradients are necessary when studying the response of different plants to graphene addition.

### 3.2. Effects of Different Graphene Addition Levels on Nutrients in Different Shrubs Soil

Graphene contains numerous oxygen-containing functional groups on its surface, which effectively adsorb nutrient ions and water in the soil. The addition of graphene to soil reduces bulk density, increases porosity, and improves soil aeration and drainage conditions, with more pronounced effects in fine-textured soils [34]. Additionally, graphene can be used as a fertilizer additive to enhance fertilizer efficiency and promote sustainable agricultural development [35]. By incorporating graphene into fertilizers, it improves soil physicochemical properties, promotes plant nutrient uptake, and reduces the chemical pollution of soil caused by fertilizers [36]. Consistent with the findings of most researchers [37], graphene addition increases soil nutrient content, primarily due to its large specific surface area and strong adsorption capacity for soil nutrient ions. This enhances soil nutrient retention, reduces nutrient leaching losses from irrigation and precipitation, and effectively increases the available nutrient content in the soil [18]. In this study, most soil nutrient indicators for the three shrub species reached their maximum values under the C2 treatment, while the total nitrogen content in *A. mongolica* soil and the available nitrogen content in *N. tangutorum* soil peaked under the C3 treatment. This may be due to the uneven distribution of soil nutrients, which results in varying adsorption capabilities of graphene in the soil [38]. Redundancy analysis indicated that soil organic matter, available nitrogen, and available phosphorus are the key factors influencing shrub growth. This is likely because the carbon molecular structure of graphene is similar to that of plants, and the introduction of a carbon source can activate soil nutrients, creating a favorable environment for soil microorganisms. This, in turn, enhances the activity of enzymes such as urease and phosphatase, promoting nitrogen and phosphorus cycling in the soil [39]. Potassium is also an essential element for plant growth, improving plant quality and increasing resistance to pests and diseases. However, due to the weak adsorption and high mobility of potassium ions, plant uptake of potassium is limited [40]. In this study, the increase in available potassium content in the soil for all shrub species was higher than that in the control, aligning with the findings of Fan Lichun [41], who discovered that the utilization rate of potassium fertilizer treated with nanocarbon was higher than that of conventional fertilizer application. This is because graphene can increase the mean weight diameter and geometric mean diameter of soil aggregates, promoting the formation of soil structure and improving soil nutrient retention, thereby enabling the slow release of fertilizer [42]. At present, the application of graphene in agriculture and forestry is still quite limited, and it is often used in combination with other materials. Therefore, selecting materials with high research value will be a critical aspect of future studies on the addition of graphene.

### 3.3. Comprehensive Analysis

Currently, data analysis methods for the comprehensive evaluation of exogenous additives include grey relational analysis, principal component analysis, and correlation analysis. Employing scientifically sound evaluation methods is crucial for determining the optimal dosage of exogenous additives [43]. In this study, 15 indicators, including shrub growth traits, biomass, and soil physicochemical properties under different graphene treatments, were analyzed using principal component analysis. The results identified total biomass as the primary indicator of the effect of graphene addition on shrub growth. Correlation analysis revealed a highly significant positive relationship (*p* < 0.01) between total biomass and the root-to-shoot ratio, available soil nitrogen, and available soil potassium. Further analysis of the linear relationship between total biomass and soil nutrients indicated that graphene promotes shrub growth by increasing the content of available nutrients in the soil. Redundancy analysis of the relationship between soil nutrients and shrub growth indicators identified soil organic matter as the key factor influencing shrub growth. Through grey relational analysis, the relational values of different graphene treatments for each shrub species were calculated and ranked, revealing that the C2 treatment was optimal for both *A. mongolica* and *X. sorbifolium*, while the C3 treatment was most effective for *N. tangutorum*. Considering the uniform distribution of graphene in the soil, cluster analysis was performed, grouping the three shrub species and five graphene treatment levels into three clusters. The C2 treatment resulted in the best overall growth and soil nutrient characteristics for *A. mongolica*, while the C4 treatment produced the poorest overall characteristics for *X. sorbifolium* and *N. tangutorum*. However, this study is limited to pot experiments investigating the effects of graphene on plant growth and soil nutrients. Further research is needed to explore the mechanisms by which graphene improves soil carbon storage and influences plant growth.

## 4. Materials and Methods

### 4.1. Research Materials

The graphene solution used in this study was provided by the Coal-Based Ecological Carbon Sink Technology Engineering Research Center of the Ministry of Education at Shanxi Datong University. The graphene solution had a concentration of 5 g/L, with graphene sheets averaging 40 nm in diameter, approximately 3 nm in thickness, and consisting of around five layers, making it multilayer graphene. The planting materials consisted of seeds of *Nitraria tangutorum*, *Xanthoceras sorbifolium*, and *Amygdalus mongolica*, collected in 2022 from the Desert Forestry Experiment Center of the Chinese Academy of Forestry. The test soil was also obtained from the first experimental field of the Desert Forestry Experiment Center and was air-dried and sieved through a 4 mm mesh. The soil was then mixed with substrate in a 3:7 ratio (Table 6).

### 4.2. Experimental Design

Plastic pots with an outer diameter of 27 cm, a base diameter of 22 cm, and a height of 30 cm, each with drainage holes, were used for the experiment. Each pot was filled with 4.5 kg of air-dried, sieved soil. On 1 June 2023, seeds of *N. tangutorum*, *A. mongolica*, and *X. sorbifolium* were soaked, and two seeds of each species were evenly sown in the pots. After sowing, 1 L of water was poured along the inner walls of each pot. Seven days later, graphene solution was added, with five graphene treatment concentrations [44,45]: C0: 0 mg/L, C1: 25 mg/L, C2: 50 mg/L, C3: 100 mg/L, and C4: 200 mg/L. The C0 treatment served as the control (CK).Each treatment was repeated 10 times, resulting in a total of 150 pots. A volume of 200 mL of the respective graphene solution was applied to each pot, and move to outdoor shelter for rain cultivation. When the plants reached the three-leaf stage, thinning was performed to retain one seedling per pot. During the cultivation period regular watering was applied (The study area belongs to summer from June to September, the climate change during the incubation period is shown in Figure 6), with 200 mL of water per pot once a week.

### 4.3. Soil Sampling Methods

From 24–30 September 2023, soil samples were collected from the pots of *N. tangutorum*, *A. mongolica*, and *X. sorbifolium*. Use micro soil drill to drill soil samples of 0–20 cm soil layer near plant stems, with three pots randomly selected from each treatment. Litter and other debris were removed from the soil, which was then air-dried, crushed, and sieved for soil chemical analysis.

### 4.4. Indicator Measurement and Methods

#### 4.4.1. Determination of Plant Growth Indicators

The height, basal diameter, leaf width, and leaf length of the shrubs were measured on the 30th, 60th, and 90th days after emergence.

Height: The natural height of each shrub in the pot was measured using a measuring tape (The measurement error is within 0.1 cm).

Leaf length, leaf width, and stem diameter: Three leaves were randomly selected from each pot, and their leaf length, leaf width, and stem diameter were measured using a vernier caliper (The measurement error is within 0.1 mm).

#### 4.4.2. Plant Biomass Measurement

Aboveground and belowground biomass: On the 90th day after emergence, biomass was measured by selecting three pots from each treatment. The shrubs were carefully washed to remove soil, and the roots were separated from the shoots by cutting at the root collar. The fresh weight of the aboveground and belowground parts was measured. The samples were then brought back to the laboratory, dry in oven at 65 °C to constant weight, and the dry weights of the aboveground and belowground biomass were recorded.

Total biomass = aboveground biomass + belowground biomass

Root-to-shoot ratio = belowground biomass/aboveground biomass

#### 4.4.3. Determination of Soil Physical and Chemical Properties

The soil Organic matter, Total nitrogen, Total phosphorus, Total potassium, Available nitrogen, Available phosphorus, Available potassiumwere measured using conventional soil agrochemical analysis methods [46], The specific measurement method is shown in Table 7.

#### 4.4.4. Comprehensive Evaluation

This study utilized SPSS 20.0 to perform principal component analysis (PCA) on 15 evaluation indicators related to shrub growth and soil nutrients under different graphene treatments. Principal components with eigenvalues ≥1 were extracted, and indicators with loadings ≥0.5 on the same principal component were grouped together. If an evaluation indicator had loadings below 0.5 on all principal components, it was assigned to the group with the highest loading value [48]. To explore the relationships between the extracted primary evaluation indicators and other indicators, Pearson correlation analysis was conducted using Origin 2022. This analysis tested the correlation between shrub growth indicators and soil nutrient indicators at significance levels of *p* < 0.01 and *p* < 0.05, with larger absolute values of the correlation coefficient indicating stronger correlations between the two variables. Additionally, to further validate the relationship between shrub growth indicators and soil nutrients, redundancy analysis (RDA) was performed using Canoco 5.0. RDA is an extension of multivariate regression models that allows the arrangement of study subjects relative to environmental factors in a defined space; the ordination axes reflect the relationships between species characteristics and environmental factors. The analysis selected seven soil factors: soil organic matter (SOM), total nitrogen (TN), total phosphorus (TP), total potassium (TK), available nitrogen (AN), available phosphorus (AP), and available potassium (AK). It also included eight plant characteristics: plant height, basal diameter, leaf length, leaf width, aboveground biomass, underground biomass, total biomass, and root-to-crown ratio. Data were centered and standardized, and the significance of the ordination axis eigenvalues was tested using Monte Carlo random simulation. In the RDA ordination plot, the length of the red arrow segments indicates the degree of influence of the soil factors; longer segments represent greater influence. The angle between species characteristics and environmental factors reflects their correlation, with angles < 90° indicating positive correlations—smaller angles suggesting stronger correlations—while angles > 90° indicate negative correlations [49]. Finally, a comprehensive evaluation was conducted using cluster analysis and grey relational analysis in SPSS 20.0 to identify the optimal graphene addition concentrations for different shrubs. The cluster analysis employed the shortest Euclidean distance method for R-type clustering of evaluation indicators, categorizing them into several groups at corresponding aggregation levels, thereby reflecting the distinct characteristics of shrub growth and soil nutrients under different graphene treatments [50]. The formula for grey relational analysis [51] is as follows:

$$\xi_{k}=\frac{{min}_{i\;}{min}_{k\;}\left|x_{0}\left(k\right)-x_{i}(k)\right|+\rho \;{max}_{i\;}{max}_{k\;}\left|x_{0}\left(k\right)-x_{i}(k)\right|}{\left|x_{0}\left(k\right)-x_{i}(k)\right|+\rho \;{max}_{i\;}{max}_{k}\left|x_{0}\left(k\right)-x_{i}(k)\right|}$$

In the formula, $x_{0}\left(k\right)-x_{i}(k)$ Indicate the absolute difference between the x_0_ and x_*i*_ sequences at point *k*, record as $\Delta_{i}(k),$ wherein ${min}_{i\;}{min}_{k}\left|x_{0}\left(k\right)-x_{i}(k)\right|$ for the second level minimum deviation, ${max}_{i\;}{max}_{k}\left|x_{0}\left(k\right)-x_{i}(k)\right|$ for the second level maximum difference, *ρ* to determine the resolution coefficient, The range of values is 0–1, Usually taken *ρ* = 0.5. Substitute the correlation coefficient of $\xi_{k}$ into the formula: $r_{i}=\frac{1}{n}\sum_{k=1}^{n}\xi_{\;(k)\;}$, Calculate the correlation value.

### 4.5. Statistical Analysis

Data processing and chart creation were performed using Excel 2010. Variance analysis was conducted using SPSS 20.0, with results expressed as mean values + standard errors. Principal component analysis, cluster analysis, and grey relational analysis were also conducted using SPSS. Correlation analysis and the generation of heat maps were performed using Origin 2022, while redundancy analysis (RDA) and related plotting were carried out using Canoco 5.0.

## 5. Conclusions

This study demonstrates that graphene addition can promote plant growth and alleviate soil infertile in arid regions. As graphene manufacturing processes continue to improve, production costs have significantly decreased, making graphene materials highly advantageous for applications in agriculture and forestry. Additionally, if we can regulate the concentration of graphene materials and clarify the tolerance dose of plants to graphene based materials, it will effectively promote a new round of technological revolution in the agriculture and forestry industry. Although the toxicity of graphene remains debated, no conclusive evidence has yet established its toxicity. It is crucial to monitor the accumulation and dispersal patterns of graphene materials in plants and the environment. By tracking their distribution, further evaluate the cumulative effect of graphene in plants and intergenerational migration risk, as well as the possible ecological risk.

## Figures and Tables

**Table 1:** The effect of different levels of graphene addition on shrub growth indicators.

Time	Treat	Heigh (cm)			Base Diameter (mm)			Leaf Width (mm)			Leaf Length (mm)		
		BT	WGG	BC	BT	WGG	BC	BT	WGG	BC	BT	WGG	BC
30	C0	8.49 ± 0.64 a	14.63 ± 0.85 b	3.58 ± 0.27 b	1.24 ± 0.05 b	1.67 ± 0.04 ab	0.80 ± 0.21 b	9.15 ± 0.27 b	5.21 ± 0.21 c	3.34 ± 0.16 a	18.33 ± 1.45 b	15.97 ± 0.52 b	14.00 ± 0.71 a
	C1	11.99 ± 2.12 a	18.80 ± 0.51 a	4.38 ± 0.55 a	1.23 ± 0.05 b	1.74 ± 0.12 ab	1.04 ± 0.04 a	8.56 ± 0.22 bc	6.18 ± 0.20 ab	3.82 ± 0.22 a	18.71 ± 0.65 b	20.67 ± 0.62 a	14.17 ± 1.08 a
	C2	13.13 ± 1.73 a	16.83 ± 1.64 ab	4.17 ± 0.40 a	1.53 ± 0.04 a	1.99 ± 0.20 a	0.94 ± 0.05 a	12.49 ± 0.82 a	7.05 ± 0.39 a	3.86 ± 0.20 a	23.60 ± 0.48 a	20.83 ± 1.66 a	15.14 ± 1.12 a
	C3	10.83 ± 0.69 a	15.67 ± 2.35 ab	4.36 ± 0.48 a	1.32 ± 0.01 b	1.79 ± 0.12 ab	0.94 ± 0.06 a	10.13 ± 0.34 b	6.48 ± 0.49 ab	3.66 ± 0.16 a	20.25 ± 1.14 b	19.33 ± 0.88 a	14.38 ± 0.60 a
	C4	11.80 ± 0.72 a	17.88 ± 0.84 ab	4.00 ± 0.54 a	1.55 ± 0.08 a	1.58 ± 0.07 b	0.94 ± 0.04 a	7.45 ± 0.57 c	5.56 ± 0.21 bc	3.32 ± 0.17 a	19.30 ± 1.45 b	14.00 ± 0.89 b	12.44 ± 1.21 a
60	C0	12.50 ± 1.85 a	17.88 ± 1.99 a	12.03 ± 0.21 a	1.50 ± 0.14 c	2.28 ± 0.08 b	0.81 ± 0.07 b	10.08 ± 0.50 a	6.88 ± 0.37 b	4.08 ± 0.19 a	22.75 ± 1.49 a	21.00 ± 0.71 ab	17.67 ± 2.85 a
	C1	14.51 ± 2.05 a	20.25 ± 1.43 a	13.32 ± 1.44 a	2.25 ± 0.08 a	2.37 ± 0.10 ab	1.16 ± 0.08 a	11.14 ± 0.26 a	8.15 ± 0.46 a	4.12 ± 0.22 a	22.00 ± 1.14 a	24.25 ± 1.11 a	19.50 ± 1.48 a
	C2	14.65 ± 2.20 a	20.15 ± 0.89 a	15.94 ± 1.63 a	1.83 ± 0.12 bc	2.36 ± 0.11 ab	1.20 ± 0.10 a	12.03 ± 2.30 a	8.46 ± 0.23 a	4.00 ± 0.31 a	25.33 ± 1.78 a	23.67 ± 0.62 a	19.53 ± 1.33 a
	C3	15.13 ± 1.31 a	19.07 ± 1.53 a	13.44 ± 1.99 a	1.99 ± 0.05 ab	2.69 ± 0.01 a	0.97 ± 0.09 ab	13.42 ± 0.20 a	6.77 ± 0.35 b	4.03 ± 0.20 a	22.33 ± 0.67 a	21.67 ± 2.40 a	19.40 ± 1.50 a
	C4	14.00 ± 2.52 a	18.94 ± 2.38 a	11.10 ± 0.59 a	1.84 ± 0.03 bc	2.52 ± 0.12 ab	1.04 ± 0.05 ab	11.61 ± 2.03 a	6.54 ± 0.12 b	3.60 ± 0.18 a	21.67 ± 0.33 a	17.00 ± 1.52 b	15.00 ± 2.00 a
90	C0	16.33 ± 0.66 a	18.08 ± 1.34 a	15.03 ± 0.77 a	1.63 ± 0.05 c	2.55 ± 0.10 a	0.99 ± 0.04 a	11.39 ± 0.30 a	7.12 ± 0.39 a	4.80 ± 0.22 a	24.00 ± 1.32 a	22.50 ± 1.19 ab	22.77 ± 0.23 a
	C1	17.88 ± 1.93 a	22.40 ± 1.32 a	16.67 ± 1.30 a	2.45 ± 0.12 a	2.61 ± 0.09 a	1.20 ± 0.15 a	11.55 ± 0.66 a	8.29 ± 0.50 a	5.00 ± 0.19 a	24.12 ± 0.96 a	25.33 ± 0.53 ab	23.72 ± 1.55 a
	C2	16.78 ± 1.70 a	22.03 ± 0.84 a	17.20 ± 1.24 a	2.04 ± 0.15 b	2.54 ± 0.12 a	1.34 ± 0.15 a	13.26 ± 1.18 a	8.79 ± 0.24 a	4.99 ± 0.35 a	31.07 ± 2.24 a	27.67 ± 1.25 a	22.32 ± 1.22 a
	C3	16.67 ± 1.96 a	21.10 ± 1.22 a	16.90 ± 1.77 a	2.10 ± 0.07 ab	2.84 ± 0.04 a	1.16 ± 0.08 a	14.62 ± 0.26 a	7.32 ± 0.40 a	5.1 ± 0.67 a	29.23 ± 3.24 a	23.63 ± 3.18 ab	23.76 ± 1.61 a
	C4	19.47 ± 1.49 a	19.40 ± 1.19 a	13.50 ± 1.95 a	2.08 ± 0.07 ab	2.74 ± 0.10 a	1.08 ± 0.03 a	12.11 ± 1.25 a	7.49 ± 0.69 a	3.89 ± 0.28 b	25.67 ± 3.21 a	19.76 ± 1.90 b	18.33 ± 2.03 a

Note: BT: *Amygdalus mongolica*, WGG: *Xanthoceras sorbifolium*, BC: *Nitraria tangutorum*. Different lowercase indicate significant differences between different treatments within the same time frame (*p* < 0.05), same as below.

**Table 2:** The effect of different levels of graphene addition on soil nutrients of smooth brome.

Plants	Treat	Organic Matter (g/kg)	Total Nitrogen (g/kg)	Total Phosphorus (g/kg)	Total Potassium (g/kg)	Available Nitrogen (mg/kg)	Available Phosphorus (mg/kg)	Available Potassium (mg/kg)
BT	C0	88.61 ± 3.71 a	2.16 ± 0.03 c	0.68 ± 0.01 a	14.81 ± 0.47 b	433.01 ± 21.47 c	41.63 ± 3.68 a	1752.50 ± 60.62 c
	C1	99.77 ± 7.18 a	2.98 ± 0.04 b	0.72 ± 0.02 a	14.89 ± 0.71 b	436.46 ± 10.55 c	50.00 ± 2.89 a	1762.50 ± 8.66 c
	C2	100.17 ± 1.37 a	3.71 ± 0.19 ab	0.68 ± 0.01 a	16.49 ± 0.08 a	592.14 ± 7.10 a	44.13 ± 2.38 a	2396.25 ± 23.82 a
	C3	105.75 ± 0.22 a	3.89 ± 0.12 a	0.68 ± 0.07 a	14.00 ± 0.08 b	511.99 ± 7.12 b	50.13 ± 1.52 a	2002.46 ± 86.60 b
	C4	99.29 ± 8.78 a	3.29 ± 0.47 ab	0.64 ± 0.01 a	14.57 ± 0.43 b	483.57 ± 22.70 bc	43.38 ± 1.80 a	1162.74 ± 37.53 d
WGG	C0	89.61 ± 5.40 a	2.83 ± 0.10 b	0.64 ± 0.10 b	15.21 ± 0.40 a	451.78 ± 72.28 b	39.13 ± 0.22 cd	1692.50 ± 135.68 b
	C1	96.98 ± 0.76 a	3.06 ± 0.04 a	0.65 ± 0.00 b	15.06 ± 0.36 a	566.38 ± 29.57 ab	43.75 ± 0.81 b	1970.00 ± 33.20 a
	C2	98.01 ± 4.00 a	3.21 ± 0.01 a	0.72 ± 0.01 a	16.14 ± 0.23 a	588.95 ± 20.64 a	45.88 ± 0.79 a	1810.00 ± 1.44 ab
	C3	95.05 ± 2.84 a	3.10 ± 0.07 a	0.65 ± 0.00 b	16.06 ± 0.53 a	523.59 ± 18.38 ab	38.88 ± 0.65 d	1890.00 ± 62.07 ab
	C4	99.64 ± 1.52 a	2.77 ± 0.09 b	0.73 ± 0.00 a	15.71 ± 0.23 a	499.90 ± 7.71 ab	41.00 ± 0.43 c	1852.50 ± 11.55 ab
BC	C0	88.46 ± 0.40 a	2.67 ± 0.01 ab	0.65 ± 0.02 ab	15.48 ± 0.43 abc	267.69 ± 25.03 b	39.00 ± 1.15 b	1250.00 ± 125.57 c
	C1	84.79 ± 3.63 a	2.63 ± 0.10 ab	0.66 ± 0.01 a	14.11 ± 0.27 c	390.60 ± 16.91 a	44.38 ± 1.66 a	1705.00 ± 36.08 ab
	C2	87.67 ± 4.05 a	2.55 ± 0.08 c	0.60 ± 0.01 ab	16.45 ± 0.33 ab	388.56 ± 18.40 a	33.50 ± 2.45 c	1842.50 ± 127.02 a
	C3	89.84 ± 1.52 a	2.93 ± 0.03 a	0.64 ± 0.02 ab	14.54 ± 1.08 bc	410.10 ± 60.80 a	38.13 ± 0.51 b	1797.50 ± 11.55 a
	C4	84.83 ± 0.57 a	2.77 ± 0.19 ab	0.61 ± 0.02 b	16.56 ± 0.5 a	254.13 ± 4.70 b	45.25 ± 0.43 a	1485.00 ± 93.82 bc

Note: Different lowercase indicate significant differences between different treatments within the same time frame (*p* < 0.05), same as below.

**Table 3:** Principal component analysis of shrub growth indicators and soil nutrients at different levels of graphene addition.

Factor	Ingredient			
	1	2	3	4
Plant heigh	0.146	−0.504	0.63	−0.069
Base diameter	0.347	−0.413	0.51	−0.373
Leaf width	0.264	0.505	0.497	0.328
Leaf length	0.255	0.476	0.379	0.368
Aboveground biomass	0.669	−0.525	0.043	−0.113
Underground biomass	0.870	−0.241	−0.145	0.287
Total biomass	0.878	−0.369	−0.088	0.163
Root shoot ratio	0.806	0.006	−0.146	0.336
Organic matter	0.596	0.555	−0.126	−0.162
Total nitrogen	0.561	0.469	−0.188	−0.256
Total phosphorus	0.468	0.244	0.258	0.161
Total Potassium	0.052	−0.397	−0.509	0.349
Available nitrogen	0.854	0.055	0.014	−0.167
Available phosphorus	0.258	0.759	−0.108	−0.28
Available potassium	0.582	−0.151	−0.176	−0.46
Characteristic value	4.928	2.724	1.518	1.179
Contribution rate (%)	32.86	18.16	10.12	7.86
Accumulated contribution rate (%)	32.86	50.12	61.13	68.99

**Table 4:** Analysis of shrub growth indicators and soil nutrient redundancy at different levels of graphene addition.

Indexes	Soil Nutrient	Contribution (%)	Explains (%)	F	*p*-Value
Plant growth	Organic matter	57.6	34.4	6.8	0.008
	Available nitrogen	16	9.5	2	0.164
	Available phosphorus	7.4	4.4	0.9	0.386
	Total nitrogen	5.1	3.1	0.6	0.502
	Total potassium	5	3	0.6	0.496
	Available potassium	5	3	0.6	0.488
	Total phosphorus	4	2.4	0.4	0.588

**Table 5:** Comprehensive analysis of grey correlation between shrub growth indicators and soil nutrients at different levels of graphene addition.

Treat	Associated Value	Sort	Treat	Associated Value	Sort	Treat	Associated Value	Sort
BT-C0	0.61	4	WGG-C0	0.67	5	BC-C0	0.55	5
BT-C1	0.68	3	WGG-C1	0.80	2	BC-C1	0.59	3
BT-C2	0.78	1	WGG-C2	0.84	1	BC-C2	0.59	2
BT-C3	0.75	2	WGG-C3	0.75	3	BC-C3	0.60	1
BT-C4	0.54	5	WGG-C4	0.73	4	BC-C4	0.55	4

**Table 6:** Basic nutrient content of experimental soil.

Organic Matter (g/kg)	Total Nitrogen (g/kg)	Total Phosphorus (g/kg)	Total Potassium (g/kg)	Available Nitrogen (mg/kg)	Available Phosphorus (mg/kg)	Available Potassium (mg/kg)
94.06	3.11	0.67	14.84	458.11	50.01	1519.38

**Table 7:** Determination and method of soil chemical index.

Measurement Indicators	Measurement Method	Deterination Basis	Specific Measurement Method
Organic matter	Measured by the K_2_Cr_2_O_7_ external heating method	LY/T1237-1999	Soil Agrochemical Analysis [47]
Total nitrogen	Measured by the semi-micro Kjeldahl method	LY/T1228-2015	
Total phosphorus	Measured by the NaOH fusion-molybdenum antimony colorimetric method	NY/T88-198	
Total potassium	Measured by the NaOH fusion-flame photometry method	NY/T87-1988	
Available nitrogen	Measured by the alkali diffusion method	LY/T1232-2015	
Available phosphorus	Measured by the NaHCO_3_ extraction-colorimetric method	LY/T1234-2015	
Available potassium	Measured by the NH_4_OAc extraction-flame photometry method	LY/T1234-2015	

## Data Availability

The data presented in this study are available on request to the corresponding author.
